# PPAR*β*/*δ* Agonism with GW501516 Increases Myotube PGC-1*α* Content and Reduces BCAA Media Content Independent of Changes in BCAA Catabolic Enzyme Expression

**DOI:** 10.1155/2023/4779199

**Published:** 2023-06-06

**Authors:** Caroline N. Rivera, Jason S. Hinkle, Rachel M. Watne, Trent C. Macgowan, Andrew J. Wommack, Roger A. Vaughan

**Affiliations:** ^1^Department of Exercise Science, High Point University, High Point, NC, USA; ^2^Department of Chemistry, High Point University, High Point, NC, USA

## Abstract

**Background:**

Type 2 diabetes is characterized by reduced insulin sensitivity, elevated blood metabolites, and reduced mitochondrial metabolism with reduced expression of genes governing metabolism such as peroxisome proliferator-activated receptor gamma coactivator 1-alpha (PGC-1*α*). PGC-1*α* regulates the expression of branched-chain amino acid (BCAA) metabolism, and thus, increased circulating BCAA in diabetics may be partially explained by reduced PGC-1*α* expression. PGC-1*α* functions in-part through interactions with peroxisome proliferator-activated receptor *β*/*δ* (PPAR*β*/*δ*). The present report examined the effects of the PPAR*β*/*δ* agonism on cell metabolism and related gene/protein expression of cultured myotubes, with a primary emphasis on determining the effects of GW on BCAA disposal and catabolic enzyme expression.

**Methods:**

C2C12 myotubes were treated with GW501516 (GW) for up to 24 hours. Mitochondrial and glycolytic metabolism were measured via oxygen consumption and extracellular acidification rate, respectively. Metabolic gene and protein expression were assessed via quantitative real-time polymerase chain reaction (qRT-PCR) and western blot, respectively. Media BCAA content was assessed via liquid chromatography–mass spectrometry (LC/MS).

**Results:**

GW significantly increased PGC-1*α* protein expression, mitochondrial content, and mitochondrial function. GW also significantly reduced BCAA content within culture media following 24-hour treatment; however, expression of BCAA catabolic enzymes/transporter was unchanged.

**Conclusion:**

These data confirm the ability of GW to increase muscle PGC-1*α* content and decrease BCAA media content without affecting BCAA catabolic enzymes/transporter. These findings suggest heightened BCAA uptake (and possibly metabolism) may occur without substantial changes in the protein levels of related cell machinery.

## 1. Introduction

Peroxisome proliferator-activated receptors (PPARs) are a nuclear receptor superfamily that include three isoforms that are differentially expressed in various tissues and regulate several aspects of cell energetics. In skeletal muscle, peroxisome proliferator-activated receptor delta (PPAR*β*/*δ*) is the predominant PPAR isoform that regulates mitochondrial content through the activation and upregulation of peroxisome proliferator-activated receptor gamma coactivator 1-alpha (PGC-1*α*) content [1]. PGC-1*α* acts as a transcriptional cofactor regulating the expression of nuclear respiratory factors (NRF1/2) [2–4] and mitochondrial transcription factor A (TFAM) [3, 5, 6], which together coordinate mitochondrial biogenesis [7]. Consistent with these observations, PPAR*β*/*δ*-deficient skeletal muscle displays reduced genes associated with fatty acid uptake and oxidation, as well as mitochondrial respiratory components, which may result from reduced regulators of mitochondrial biogenesis including PGC-1*α* and TFAM [8].

In addition to insulin resistance and elevated circulating glucose, reduced PGC-1*α* expression in metabolically meaningful tissues also occurs in the diabetic phenotype [9]. Moreover, like many circulating metabolites that are elevated during metabolic disease (such as glucose and lipids), amino acids, specifically the branched-chain amino acids (BCAA), have been consistently correlated with severity of insulin resistance [10–13]. One possible mechanism linking insulin resistance to increased circulating BCAA is that metabolically diseased populations may experience diminished BCAA catabolic capacity [14]. Thus, it is conceivable BCAA may accumulate in the blood of those with diabetes due to reduced BCAA metabolism. Interestingly, the BCAA degradation enzymes, branched-chain alpha-keto acid dehydrogenase (BCKDH) and branched-chain aminotransferase (BCAT) [15], are controlled by both the PPARs and PGC-1*α* [16–19]. In line with these observations, Lian et al. showed activation of myotube 5′-AMP-activated protein kinase (AMPK), a master regulator of energetics which activates PGC-1*α* [20], appears to increase BCAA catabolic enzyme expression and/or activity [21]. Importantly, however, activation of BCAA catabolism via AMPK activators, such as AICAR during cell culture experiments, might be dependent on concentration and glucose availability [22], and may reduce BCAA catabolism despite an upregulation of BCAA catabolic enzymes, which may accompany metabolic stress as seen with atrophy [23, 24]. Collectively, these observations agree with others demonstrating exercise (another activator of PPAR*β*/*δ*, AMPK, and PGC-1*α*) increases BCAA metabolism [15]. Taken together, it is conceivable the activation of PPAR*β*/*δ*, which works synergistically with AMPK and PGC-1*α*, represents a potential method of improving mitochondrial function (and BCAA metabolism), which is often suppressed during insulin resistance.

Several past reports have shown the selective PPAR*β*/*δ* agonist, GW501516 (GW), can activate PPAR*β*/*δ* and PGC-1*α* leading to improved mitochondrial function. For example, L6 myotubes treated with GW exhibited increased palmitate oxidation and PGC-1*α* expression, as well as consistently elevated mRNA expression of indicators of lipid oxidation [1]. The effect of GW on palmitate oxidation was also observed in C57BL/6J mice, as were elevations in PGC-1*α* expression, lipid oxidation enzymes, and uncoupling protein mRNA expression [1]. Not surprisingly, GW-treated mice displayed increased skeletal mitochondrial content in both chow- and high-fat-fed mice. Thus, it was concluded that PPAR*β*/*δ* activation upregulates both PGC-1*α* expression and mitochondrial biogenesis both *in vitr*o and *in vivo* [1]. Other reports have found similar results. For example, treatment with GW and BMS649 (a selective retinoid X receptor (RXR) agonist) induced PGC-1*α* expression in C2C12 myocytes [8]. Another report showed C2C12 myoblasts treated for 24 hours with 1 *μ*M GW displayed increased PGC-1*α* promoter activity and expression [25]. Similarly, C2C12 myotubes treated with GW for 24 hours displayed increased PGC-1*α* and carnitine palmitoyl transferase 1 b (*Cpt1b*) mRNA, which was PPAR*β*/*δ*-dependent (not induced by PPAR*α* activation). The same report showed PGC-1*α* also controlled PPAR*β*/*δ* expression. Moreover, L6 myotubes treated with GW for 24 hours showed increased palmitate oxidation, CPT1b, and cytochrome c oxidase expression [26]. GW at 5 *μ*M has been shown to increase oleic acid oxidation and *Cpt1* mRNA expression [27]. And in mice with myocardial infarction, those treated with GW also exhibited increased oleic acid oxidation and enhanced *Cpt1* mRNA expression, but not PGC-1*α* expression, which corresponded to increased running duration and distance [27]. Another report also demonstrated myotubes treated with GW displayed increased *Cpt1* mRNA expression; however, GW-treated cells displayed unaltered mitochondrial respiration but increased proton leak [28]. Interestingly, primary myotubes exposed to GW for 6 hours displayed unaltered *Ppargc1a* and reduced *Ppard* mRNA expression; however, GW-treated cells also displayed enhanced glucose uptake [29].

Together, much of the available data suggest PPAR*β*/*δ* agonism via GW efficiently and reliably activates PGC-1*α* expression (as well as related downstream targets). However, to our knowledge, no report has also simultaneously investigated the effects of PPAR*β*/*δ* agonism using GW on metabolism as well as regulators of BCAA catabolism in muscle. Because PGC-1*α* has been shown to regulate BCAA catabolic enzymes [16, 19, 30] (which is reduced in those with diabetes [9]), we sought to determine if PPAR*β*/*δ* agonism via GW would alter cell metabolism as well as indicators of BCAA catabolism in a model of skeletal muscle.

## 2. Materials and Methods

### 2.1. Cell Culture

C2C12 mouse myoblasts from ATCC (Manassas, VA) were cultured in Dulbecco's Modified Eagle's Medium (DMEM) containing 4500 mg/L glucose and supplemented with 20% heat-inactivated fetal bovine serum (FBS), 100 U/mL penicillin, and 100 *μ*g/mL streptomycin in a humidified 5% CO_2_ atmosphere at 37°C. Cells were grown to confluency with growth media changed every 2–3 days (using cell passages <15 for all experiments). Differentiation was accomplished by replacing growth media with DMEM supplemented with 2% horse serum, 100 U/mL penicillin, and 100 *μ*g/mL streptomycin for 6–8 days. Stock GW501516 (GW) from Enzo Life Sciences (Farmingdale, NY) was dissolved in dimethylsulfoxide (DMSO) to a concentration of 1 mM. Cells were then treated with either differentiation media containing equal volume DMSO (0.1% vol/vol), which served as the control condition, or GW at 1 *μ*M (also containing 0.1% vol/vol DMSO) for up to 24 hours, which has been shown to be sufficient for increasing PPAR*β*/*δ* activity by previous observations [25, 26, 28, 31].  Table 1 summarizes GW treatment doses and durations from similar reports. To discern the potential mechanistic involvement of PPAR*β*/*δ*, we repeated experiments with and without GSK3787 (GSK), a selective PPAR*β*/*δ* antagonist [32, 33], at 10 *μ*M (also using 0.1% vol/vol DMSO). Therefore, during inhibition experiments with GSK, DMSO volume was increased and normalized to 0.2% vol/vol for all groups. To further investigate the effects of GW on BCAA metabolism during insulin resistance, we also included insulin resistant groups during select experiments. Insulin resistance was induced by the addition of insulin 100 nM for the final 3 days of differentiation [34–38], which significantly reduces insulin-mediated insulin receptor activation, insulin-mediated insulin receptor substrate 1 (IRS1) activation, insulin-mediated phosphatidylinositol-3 kinase (PI3K) activation, and insulin-mediated glucose uptake without altering cell differentiation status [37, 38]. In addition to these targets, Akt is a target of insulin downstream of IRS1 and PI3K, which is regarded as a central node in the proximal insulin signaling cascade [39]. This model of insulin resistance has been shown to dose-dependently reduce Akt activation following insulin stimulation [36, 40], and was therefore used in the current report. Additionally, we have previously shown that this model of insulin resistance leads to the extracellular accumulation of BCAA within media following a single day recovery with stock media (composition available at the following product link: 12-614-Lonza Bioscience) [41]. Thus, cells were treated with and without both GW and/or GSK in stock media following the induction of insulin resistance or not.

### 2.2. Cell Viability and Nuclei Content

**Figure 1:**
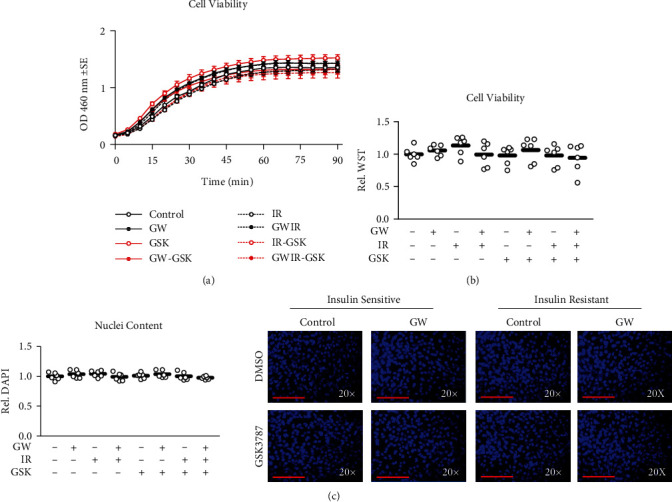
Cell viability. (a and b) Effect of GW501516 (GW) at 1 *μ*M for 24 hours both with and without GSK3787 at 10 *μ*M both with and without insulin resistance presented as (a) WST-1 assay time trial or (b) final relative measurement at 90 minutes. (c) Relative nuclei content from cells treated as described in “a” with representative images presented at right. Notes: Data were analyzed using one-way ANOVA with Bonferroni's correction for multiple comparisons. Measurements were performed using *n* = 3 individual replicates per treatment condition and were repeated across two independent experiments with *n* = 6 per group in the final analyses using the average of three measurements per experiment. Representative images from DAPI staining were taken using the 20× objective with red line indicating 150 *μ*m.

Following treatment as described above, media was replaced with media supplemented with Water-Soluble Tetrazolium 1 (WST-1) substrate. Cells/media supernatant were immediately measured for absorbance at 450 nm temporally for 90 minutes. Cell viability did not differ between any groups (Figures 1(a) and 1(b)). In separate experiments, media was collected following treatment as described above, and cells were fixed using media supplemented with 3.7% formaldehyde at 37°C with a 5% CO_2_ atmosphere. The fixing agent was then removed, and cells were stained with 4′,6-diamidino-2-phenylindole (DAPI) at 0.5 *μ*M in phosphate buffered saline (PBS), incubated in the dark at room temperature for 10 minutes, and fluorescence was measured at 360/460 nm, which did not significantly differ between groups (Figure 1(c)). Both experiments were performed using two independent experiments each comprised of four replicates per group for *n* = 6 in the final analyses. Measurements were performed in triplicate, and the average was used for both outcomes.

### 2.3. Quantitative Real-Time Polymerase Chain Reaction

**Figure 2:**
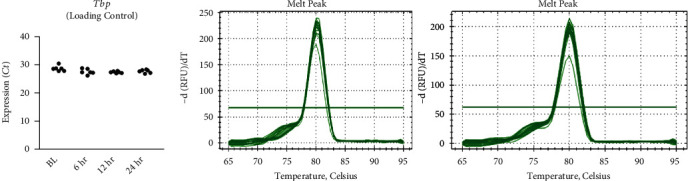
Effect of GW501516 on *Tbp* Ct values from qRT-PCR experiments. Notes: Time course gene expression was analyzed using one-way ANOVA with Bonferroni's correction for multiple comparisons with no significant pair-wise comparisons identified. Tata binding protein (*Tbp*) values were measured using three replicates per group across two independent experiments with *n* = 6 for the final analysis.

To investigate the effect of GW on protein expression of related metabolic targets, cells were differentiated and treated as described above. Total mRNA was extracted using the Trizol method and quantified (via NanoDrop from Thermo Fisher, Wilmington, DE), and cDNA was synthesized using the iScript cDNA Synthesis Kit from Bio-Rad (Hercules, CA) according to manufacturer's instructions. Polymerase chain reaction (PCR) primers were synthesized by Integrated DNA Technologies (Coralville, IA) (Table 2). Amplification of target genes was normalized to the housekeeping gene, TATA binding protein (*Tbp*), which did not significantly differ between groups (Figure 2). Quantitative real-time polymerase chain reactions (qRT-PCR) were performed using the CFX Connect System from Bio-Rad (Hercules, CA). SYBR Green-based PCR was performed using final primer concentrations at 3.75 *μ*M in a total volume of 10 *μ*l per well. The following cycling parameters were used: 95°C for 3 minutes followed by 40 cycles of 95°C for 15 seconds, and 60°C for 30 seconds. qRT-PCR was performed using *n* = 3 per treatment condition from two independent experiments with *n* = 6 for the final analysis. Relative quantification was determined via *ΔΔ*Ct method.

### 2.4. Immunoblotting

To investigate the effect of GW on protein expression of related metabolic targets, cells were differentiated and treated as described above. Whole cell lysates were then prepared by harvesting the cells on ice in Radioimmunoprecipitation Assay (RIPA) buffer supplemented with protease inhibitor mix (0.1%), followed by incubation on ice for 60 minutes. Insoluble material was removed, and protein concentrations were determined by Bradford assay. Total protein (50 *μ*g per sample) was size-separated by 10% sodium dodecyl sulfate–polyacrylamide gel electrophoresis (SDS-PAGE) and electro-transferred to polyvinylidene difluoride (PVDF) membranes. After blocking in tris-buffered saline with tween (TBST) with 5% non-fat milk powder for 1 hour, membranes were probed at 4°C overnight with antibodies, as described in Table 3, at a dilution of 1 : 1000 in TBST-5% non-fat milk powder (with the exception of large amino acid transporter 1 [LAT1] for which a dilution of 1 : 500 was used). Relative signal intensities were normalized to *β*-actin (or total target for phosphorylation of BCKDHa) and quantified using Image Lab from Bio-Rad (Hercules, CA). Bound antibodies were detected by horseradish peroxidase-conjugated secondary antibodies from AbCam (Cambridge, MA) at a dilution of 1 : 5000 in TBST-5% non-fat milk powder for 1 hour at room temperature while shaking. Protein signal intensities were determined by chemiluminescence using the Clarity Western ECL substrate kit from Bio-Rad (Hercules, CA) and imaged using the ChemiDoc Touch from Bio-Rad (Hercules, CA). Blots were performed using three replicates per condition performed across two independent experiments with *n* = 6 for the final analysis. Each target was also measured in duplicate for each experiment, and the average was used in the final analyses. Molecular weights for all targets were verified against sizes suggested by product brochures (Table 3).

### 2.5. Seahorse Metabolic Assays

For MitoStress assays, cells were seeded into Seahorse XFe96 culture plates and differentiated for 6 days once reaching confluence. Following treatment with GW for 24 hours, media was replaced with XF Assay Media obtained from Agilent Technologies (Santa Clara, CA) containing glucose at 25 mM, pyruvate at 1 mM, and glutamine at 2 mM. Following incubation, baseline measurements of oxygen consumption rate (OCR) and extracellular acidification rate (ECAR) were recorded as indicators of basal oxidative metabolism and glycolytic metabolism, respectively. Following basal measurements, each well was infused with oligomycin (an inhibitor of ATP synthase) at a final concentration of 2 *μ*M to induce maximal glycolytic metabolism. Cells were then exposed to carbonyl cyanide p-[trifluoromethoxy]-phenyl-hydrazone (FCCP) at 2 *μ*M to uncouple electron transport and induce peak OCR. Maximal respiration measurements were followed by the injection of rotenone at 1 *μ*M to reveal non-mitochondrial respiration. Basal and peak oxidative metabolism were calculated by subtracting non-mitochondrial OCR from each respective well. The Seahorse XFe96 Analyzer was run using a 6-minute cyclic protocol command (mix for 3 minutes and measure for 3 minutes). MitoStress assays included *n* = 23 per group repeated with two independent experiments for *n* = 46 per group for the final analysis. No wells presented with negative OCR values or lack of response to injection.

### 2.6. Nuclear, Mitochondrial, and Nile Red Staining

**Figure 3:**
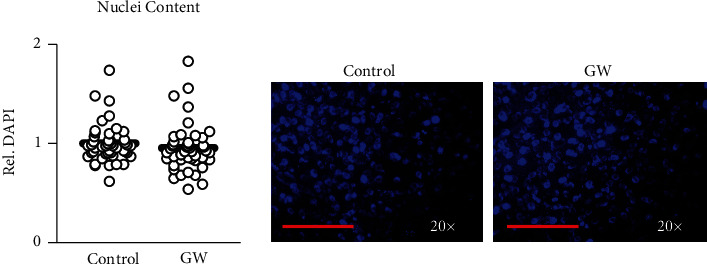
Cell content during Seahorse assay. Effect of GW501516 (GW) at 1 *μ*M for 24 hours from Seahorse metabolic assay on relative nuclei content. Data were analyzed using student's *t*-test performed using *n* = 23 individual replicates per treatment condition and repeated across two independent experiments with *n* = 46 per group in the final analyses. Representative images were taken using the 20× objective with red line indicating 150 *μ*m.

Immediately following the Seahorse metabolic assay as described above, cells were fixed using 3.7% formaldehyde at 37°C with a 5% CO_2_ atmosphere. The fixing agent was then removed, and cells were stained with DAPI at 0.5 *μ*M in PBS, incubated in the dark at room temperature for 10 minutes, and fluorescence was measured at 360/460 nm, which did not significantly differ between groups (Figure 3). Next, to assess mitochondrial content, cells were stained with 100 *μ*M nonyl acridine orange (NAO) (Fremont, CA) in PBS and incubated in the dark at room temperature for 10 minutes. Fluorescence was then measured using 485/525 nm excitation/emission and quantified in triplicate and the average (less background). Lastly, to measure lipid content, cells were fixed as described above and stained with Nile Red in PBS at 10 *μ*M Nile Red (1% DMSO vol/vol) and incubated at room temperature in the dark for approximately 5 minutes. Fluorescence was measured using 530/645 nm excitation/emission and quantified in duplicate, and the average (less background) was used in analysis. Each fluorescent measurement was performed using *n* = 23 per group repeated with two independent experiments with *n* = 46 per group for the final analyses, with each measurement performed in triplicate. Following fluorescent quantification, cells were imaged at 20× using the Motic AE31E inverted microscope and Moticam Pro 252B (Causeway Bay, Hong Kong).

### 2.7. BCAA Media Content and Liquid Chromatography–Mass Spectrometry

Using a modified protocol similar to previous measurements of BCAA in human plasma [49], media BCAA content was assessed. For experiments with both insulin resistance and GSK3787 inhibition, cells were fixed immediately following media collection using 3.7% formaldehyde at 37°C with a 5% CO_2_ atmosphere. The fixing agent was then removed, and cells were stained with DAPI at 0.5 *μ*M in PBS, incubated in the dark at room temperature for 10 minutes, and fluorescence was measured at 360/460 nm, which did not significantly differ between groups (Figure 1). Chromatographic separation and quantification of leucine, isoleucine, and valine were performed using a Shimadzu Nexera UHPLC system equipped with a Phenomenex Kinetex C18 100 Å column (100 × 3 mm, 2.6 *μ*m) kept at a temperature of 40°C connected to Shimadzu LCMS-8045 triple quadrupole mass spectrometer (Shimadzu, Kyoto, Japan) fitted with a dual ion source (DUIS) . The source used nebulizer gas 2.0 L/min, drying gas 10.0 L/minute, desolvation line (DL) temperature 250°C, and heat block temperature 400°C, with collision-induced dissociation (CID) gas 230 kPa. The mobile phases of A (water with 0.1% formic acid) and B (methanol 0.1% formic acid) were used at a flow rate of 0.4 mL/minute for the following gradient method: 0 minutes, 20% B; 1.5 minutes, 20% B; 1.7 minutes, 40% B; 3.5 minutes, 40% B; 5 minutes, 65% B; 8 minutes, 65% B; followed by 4 minutes 20% B for column equilibration. The injection volume was maintained at 1 *μ*L. This afforded reproducible retention time values for valine (1.112 minutes), isoleucine (1.356 minutes), and leucine (1.425 minutes).

Shimadzu LabSolution software version 5.97 was used to acquire and process the data. The fragmentation for each BCAA was optimized using multiple-reaction monitoring (MRM) set to positive mode for valine (118.1 to 72.2 *m*/*z*, *Q*1 −23.0 V, CE −12.0 V, and *Q*3 −20.0 V), isoleucine (132.0 to 69.2 *m*/*z*, *Q*1 −10.0 V, CE −19.0 V, and *Q*3 −11.0 V), and leucine (132.1 to 43.2 *m*/*z*, *Q*1 −10.0 V, CE −26.0 V, and Q3 −18.0 V), with a dwell time of 100 ms.

**Figure 4:**
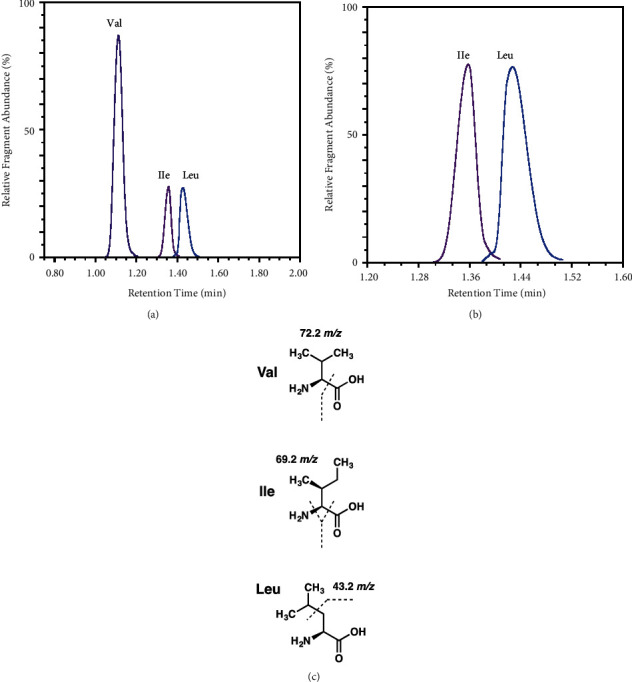
MRM chromatograms and plausible BCAA fragmentations. (a) Separation of BCAAs was achieved with UHPLC gradient to deliver distinct retention times of the product ions following optimized collision energies. (b) The chromatographically resolved isomeric analytes of Ile and Leu could be further distinguished by producing two unique fragments at *m/z* 69.2 and 43.2, respectively. (c) Suggested fragmentation sites for BCAAs.

A stock solution containing all BCAAs at a concentration of 8.0 mM was obtained by dissolving each amino acid in water/methanol solution (50 : 50, *v/v*) and kept at 4°C. Further dilutions with water/methanol were performed to assemble a calibration curve ranging from 3.125 to 100.0 *μ*M. MRM chromatograms and plausible BCAA fragmentations are shown in Figure 4.

### 2.8. Statistical Analyses

Data are presented as dot plots or group mean ± SE. Protein expression from 24-hour experiments was analyzed using student's *t*-test. Time course gene expression was analyzed using one-way ANOVA with Dunnett's correction for multiple comparisons. Metabolism time trials were analyzed via two-way ANOVA with Bonferroni's correction for multiple comparisons. Mitochondrial metabolism, mitochondrial and lipid content, as well as BCAA media content for experiments only including control and GW groups were analyzed using student's *t*-test. Total cell metabolism was analyzed using a one-way MANOVA using Wilks' Lambda, with treatment as the independent variable and mitochondrial respiration and glycolytic metabolism as the two dependent variables. BCAA media content, nuclei content, and viability for experiments with and without PPAR*β*/*δ* inhibitor both with and without insulin resistance were analyzed initially with factorial-ANOVA followed by pair-wise comparisons with Bonferroni's correction. Values of *p* ≤ 0.05 were used to identify significant differences between groups.

## 3. Results

### 3.1. GW Increases Cell Metabolism, Mitochondrial Content, and PGC-1*α* Expression

**Figure 5:**
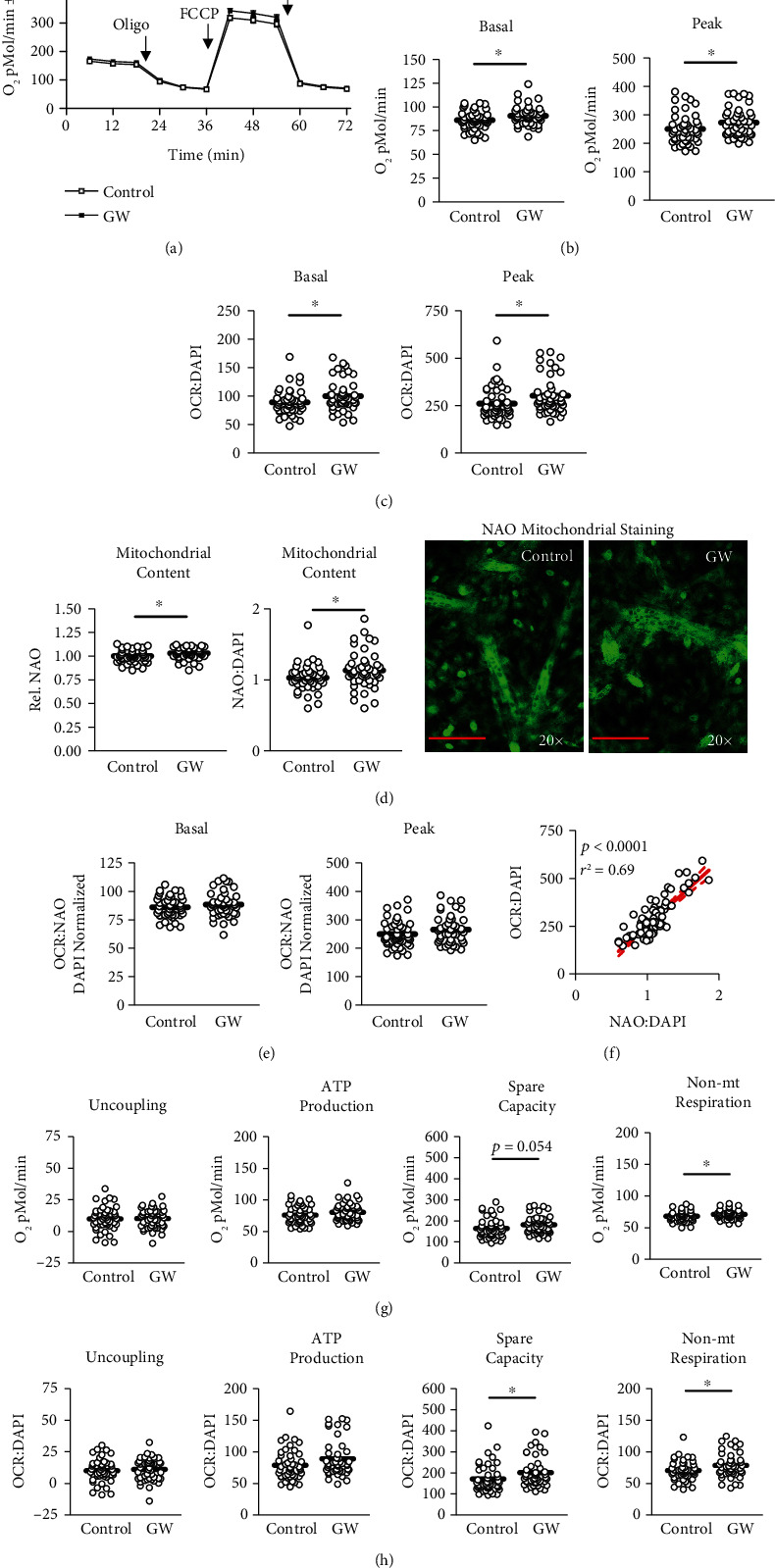
Effect of GW501516 on mitochondrial metabolism and content. (a) Time trial of mitochondrial metabolism following treatment with and without GW501516 (GW) at 1 *μ*M for 24 hours. (b and c) Basal and peak mitochondrial metabolism following treatment described in “a” presented as (b) raw values or (c) normalized to nuclei content, which did not differ between groups (see Figure 3). (d) Effect of GW at 1 *μ*M for 24 hours on mitochondrial staining both without and with normalization to nuclei content, with representative images at right. (e) Nuclei-normalized basal and peak mitochondrial metabolism following treatment described in “c” normalized to mitochondrial staining described in “d”. (f) Relationship between peak mitochondrial function and content as analyzed above presented as peak mitochondrial function normalized to nuclei content versus mitochondrial staining normalization to nuclei content. (g and h) Effect of GW at 1 *μ*M for 24 hours on mitochondrial proton leak (uncoupling), ATP production, mitochondrial spare respiratory capacity, and non-mitochondrial (non-mt) respiration, both without and with normalization to DAPI, respectively. Notes: ∗ indicates *p* ≤ 0.05 between groups. States of mitochondrial metabolism, as well as mitochondrial staining, were analyzed using student's *t*-test. States of mitochondrial metabolism were calculated by subtracting non-mitochondrial respiration from basal and FCCP-induced peak mitochondrial oxygen consumption (OCR). Time course of mitochondrial function was analyzed using two-way ANOVA with Bonferroni's correction for multiple comparisons. Metabolic analyses were performed using *n* = 23 individual replicates per treatment condition and repeated across two independent experiments with *n* = 46 per group in the final analyses. Mitochondrial staining was performed using *n* = 23 individual replicates per treatment condition and was repeated across two independent experiments with *n* = 46 per group in the final analyses using the average of three measurements per experiment. Images in “d” of representative individual myotubes were taken using the 20× objective with red line indicating 150 *μ*m. Relationship between peak mitochondrial metabolism and mitochondrial content was analyzed using linear regression. Metabolic calculations were performed as follows: Basal Respiration = Measurement #3 − Measurement #12; Peak Respiration = Measurement #7 − Measurement #12; Proton Leak = Measurement #average of 4–6 − Measurement #12; Spare Respiratory Capacity = Measurement #7 − Measurement #3; ATP-Linked Respiration = Measurement #3 − Measurement #4–6; Non-Mitochondrial Respiration = Measurement #12. Other Abbreviations: Oligo: oligomycin; FCCP: carbonyl cyanide *p*-[trifluoromethoxy]-phenyl-hydrazone; and Rot: rotenone.

**Figure 6:**
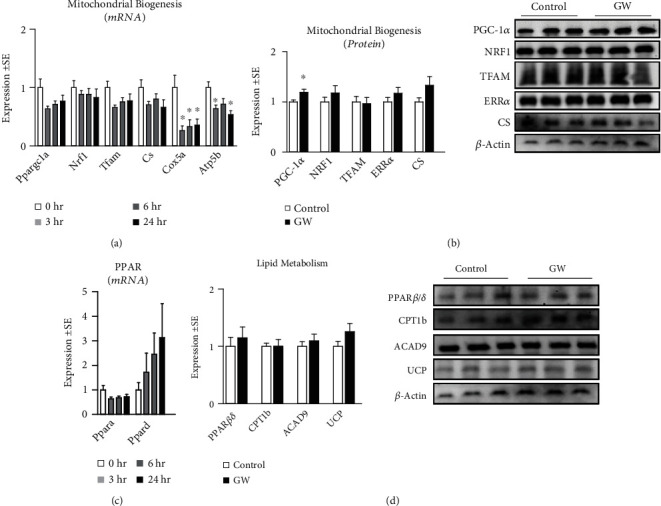
Effect of GW501516 on mitochondrial biogenic signaling. (a) Effect of treatment with GW at 1 *μ*M for various durations on myotube mRNA expression of peroxisome proliferator-activated receptor gamma coactivator 1-alpha (*Ppargc1a*), nuclear respiratory factor 1 (*Nrf1*), mitochondrial transcription factor A (*Tfam*), citrate synthase (*Cs*), cytochrome c oxidase subunit 5a (*Cox5a*), and ATP synthase subunit 5b (*Atp5f1b*). (b) Effect of treatment with GW at 1 *μ*M for 24 hours on myotube protein expression of peroxisome proliferator-activated receptor gamma coactivator 1-alpha (PGC-1*α*), nuclear respiratory factor 1 (NRF1), mitochondrial transcription factor A (TFAM), estrogen-related receptor alpha (ERR*α*), and citrate synthase (CS). (c) Time trial of the effect of treatment with GW at 1 *μ*M for up to 24 hours on myotube mRNA expression of peroxisome proliferator-activated receptor alpha (Ppara) and peroxisome proliferator-activated receptor delta (Ppard). (d) Effect of treatment with GW at 1 *μ*M for 24 hours on myotube protein expression of peroxisome proliferator-activated receptor beta (PPAR*β/δ*), carnitine palmitoyl transferase (CPT1b), acyl-coA dehydrogenase 9 (ACAD9), and uncoupling protein 1/2/3 (UCP).

**Figure 7:**
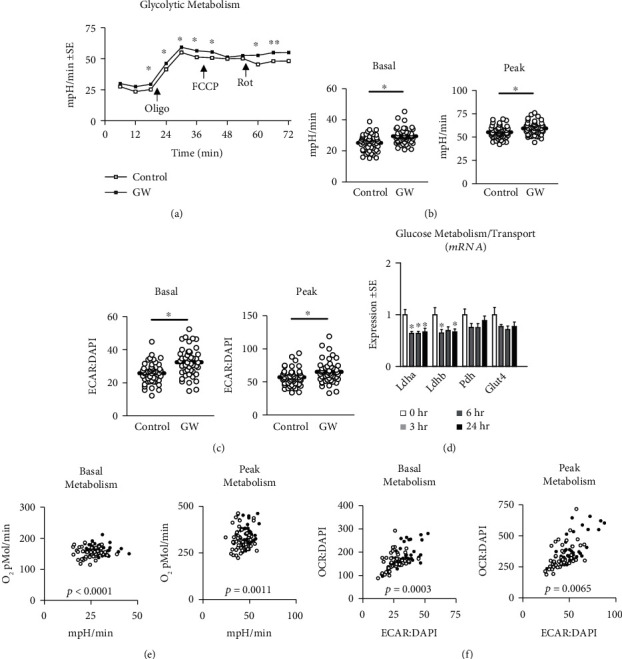
Effect of GW501516 on glycolytic metabolism, glycolytic gene expression, and total cell metabolism. (a) Time trial of glycolytic metabolism following treatment with GW501516 (GW) at 1 *μ*M for 24 hours. (b and c) Basal and peak glycolytic metabolism following treatment described in “a” presented as (b) raw values or (c) normalized to nuclei content, which did not differ between groups (see Figure 3). (d) Time trial of the effect of GW on mRNA expression of lactate dehydrogenase a (*Ldha*), lactate dehydrogenase b (*Ldhb*), pyruvate dehydrogenase (*Pdh*), and glucose transporter 4 (*Glut4*). (e and f) Basal and peak cell metabolism following treatment described in “a” (e) raw values or (f) normalized to nuclei content, which did not differ between groups (see Figure 3). Notes: Basal and peak glycolytic metabolism were analyzed using student's *t*-test. Analyses were performed using *n* = 23 individual replicates per treatment condition and were repeated across two independent experiments with *n* = 46 per group in the final analyses. Target gene expression was normalized to tata binding protein (*Tbp*) using three replicates per group across two independent experiments with *n* = 6 for the final analysis. Cell metabolism was analyzed using a one-way MANOVA using total oxygen consumption under basal or FCCP-induced peak mitochondrial respiration and corresponding glycolytic metabolism at that time point during the time trial. Other Abbreviations: Oligo: oligomycin; FCCP: carbonyl cyanide p-[trifluoromethoxy]-phenyl-hydrazone; and Rot: rotenone.

**Figure 8:**
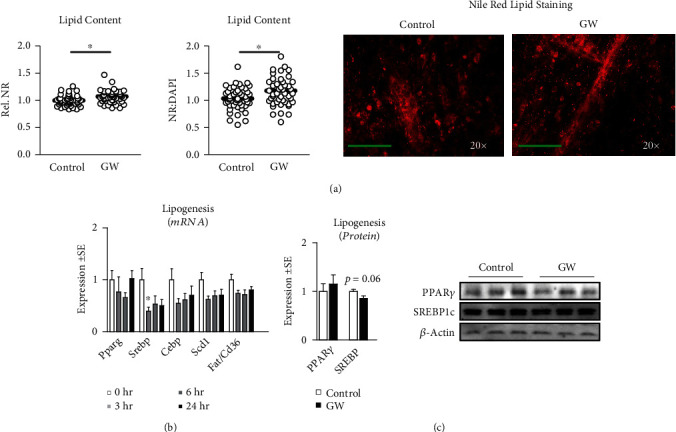
Effect of GW501516 on lipid content and related molecular targets. (a) Effect of GW at 1 *μ*M for 24 hours on lipid content (indicated by Nile red staining) both without and with normalization to nuclei content, with representative images at right. (b) Time trial of the effect of GW on mRNA expression of peroxisome proliferator-activated receptor gamma (*Pparg*), sterol regulatory element-binding protein (*Srebp*), CCAAT/enhancer-binding protein alpha (*Cebpa*), stearoyl-CoA desaturase (Scd1), and fatty acid translocase (*Fat* or *CD36*). (c) Effect of treatment with GW at 1 *μ*M for 24 hours on myotube protein expression of peroxisome proliferator-activated receptor gamma (PPAR*γ*) and sterol regulatory element-binding protein (SRECP1c). Notes: ∗ indicates *p* ≤ 0.05 between groups. Lipid staining was performed using *n* = 23 individual replicates per treatment condition repeated across two independent experiments with *n* = 46 per group in the final analysis. Images in panel “a” are representative individual myotubes imaged using the 20× objective with green line indicating 150 *μ*m. Time course gene expression was analyzed using one-way ANOVA with Dunnett's correction for multiple comparisons. Target gene expression was normalized to tata binding protein (*Tbp*) using three replicates per group across two independent experiments with *n* = 5–6 for the final analysis. Student's *t*-test was used to assess differences in metabolism, lipid content, and protein expression following 24-hour treatment. Western blots were performed using three replicates per group across two independent experiments with each target quantified in duplicate for each sample, with *n* = 6 for the final analysis.

We began our investigation by measuring the effect of GW-mediated PPAR*β*/*δ* agonism on mitochondrial metabolism (Figures 5(a), 5(b), and 5(c)) and content (Figure 5(d)) following 24-hour treatment. Both basal and peak mitochondrial respiration were significantly increased in GW-treated cells (Figures 5(a), 5(b), and 5(c)). This led us to assess mitochondrial content, which was also increased following GW treatment (Figure 5(d)). Interestingly, after normalizing mitochondrial function to mitochondrial content, changes in mitochondrial function were returned to values statistically similar to control cells, suggesting improvements in mitochondrial function were proportional (and perhaps resultant from) increases in mitochondrial content (Figure 5(e)). And not surprisingly, when analyzed via linear regression, mitochondrial staining closely predicted peak mitochondrial function (Figure 5(f)). We also assessed the effect of GW on other characteristics of mitochondrial metabolism and observed unchanged proton leak and ATP production following treatment regardless of normalization to nuclei content (Figures 5(g) and 5(h)). Conversely, spare capacity and non-mitochondrial respiration were significantly increased in GW-treated cells both without and with controlling for nuclei content (Figures 5(g) and 5(h), respectively). Next, we investigated gene expression of several regulators of mitochondrial biogenesis. Expressions of *Cox5a* and *Atp5b* were significantly reduced by GW at 3, 6, and 24 hours, and *Atp5b* was significantly reduced at 3 and 24 hours; however, no changes were observed in the expression of *Ppargc1a*, *Nrf1*, *Tfam*, or *Cs* (Figure 6(a)). Despite a lack of change at the mRNA level, PGC-1*α* was significantly increased at the protein level, while protein expression of other related factors remained unaltered (Figure 6(b)). Next, we assessed if GW treatment altered indicators of lipid metabolism. GW treatment had no effect on *Ppara* or *Ppard* mRNA expression (Figure 6(c)). Similarly, there were no changes in protein expression of markers of lipid metabolism, including PPAR*β*/*δ*, CPT1, ACAD9, and UCP1/2/3 (Figure 6(d)). Next, we assessed the effect of GW on glycolytic metabolism. In-line with the observation that PPAR*β*/*δ* agonism increases metabolism, we observed significantly increased basal and peak glycolytic metabolism in GW-treated cells (Figures 7(a), 7(b), and 7(c)). Surprisingly, this increase in glycolytic function occurred despite significant reductions in *Ldha* and *Ldhb* (but not *Pdh* or *Glut4*) expression (Figure 7(d)). Collectively, simultaneously increased basal glycolytic and mitochondrial metabolism suggests increased basal metabolism. Similarly, simultaneously increased peak glycolytic and mitochondrial metabolism suggests an overall increase in total metabolic capacity. Importantly, these changes were observed both with and without normalization to nuclei content, which did not differ between groups within the metabolic experiments (Figure 3). Interestingly, despite increased cell metabolism, GW-treated cells displayed subtly but significantly increased lipid content (Figure 8(a)); however, GW treatment reduced *Srebp* expression at 3 hours (Figure 8(b)). At the protein level, SREBP was reduced following 24 hours of GW treatment, albeit not significantly (*p* = 0.06, Figure 8(c)). Together, these findings confirm the ability of GW to enhance cell metabolism.

### 3.2. GW Reduces BCAA Media Content Independent of BCAA Catabolic Enzyme Expression

**Figure 9:**
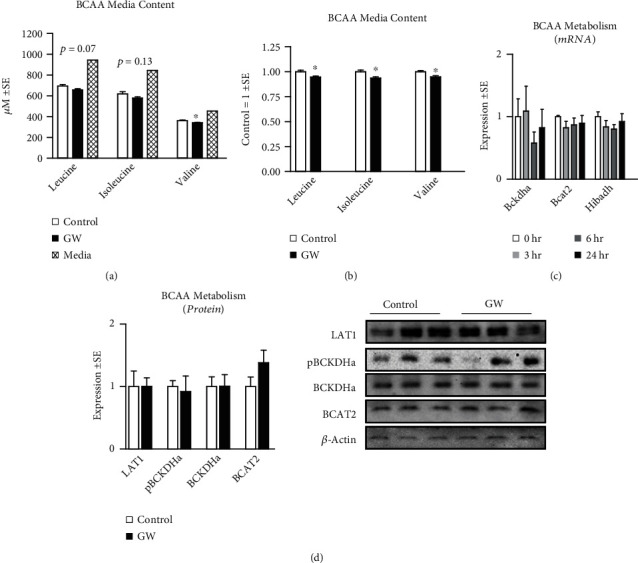
Effect of GW501516 on BCAA transport and catabolic enzymes. (a and b) Effect of treatment with GW501516 (GW) at 1 *μ*M for 24 hours on (a) absolute media BCAA content or (b) control mean-normalized (within each experiment) media BCAA content following 24-hour treatment. (c) Effect of GW at 1 *μ*M for up to 24 hours on myotube mRNA expression of branched-chain aminotransferase 2 (*Bcat2*), branched-chain alpha-keto acid dehydrogenase (*Bckdha*), and 3-hydroxyisobutyrate dehydrogenase (*Hibadh*). (d) Effect of GW at 1 *μ*M for 24 hours on myotube protein expression of large amino acid transporter 1 (LAT1), pBCKDHa (normalized to total BCKDHa), BCKDHa, and BCAT2. Notes: ∗ indicates *p* ≤ 0.05 between groups. Time course gene expression was analyzed using one-way ANOVA with Dunnett's correction for multiple comparisons. Target gene expression was normalized to tata binding protein (*Tbp*) using three replicates per group across two independent experiments with *n* = 5–6 for the final analysis. Protein expression and BCAA media content were analyzed using student's *t*-test. Western blots were performed using three replicates per group across two independent experiments with *n* = 6 for the final analysis. BCAA media content was performed using three replicates per group across two independent experiments with *n* = 6 for the final analysis with each analyte measured in triplicate.

Because we observed increased PGC-1*α* content, which is known to regulate amino acid catabolic enzyme expression [16, 19], we next measured the effect of GW on BCAA content of media following treatment with either control (DMSO) or GW for 24 hours. Consistent with our hypothesis, GW treatment reduced the absolute concentration of each individual BCAA, with significant reductions in valine (Figure 9(a)). Additionally, after normalizing groups to experimental control averages, GW treatment resulted in significant reductions (reduced by roughly 5–6%) in each of the individual BCAA versus control cells (Figure 9(b)). Given we observed significant reductions in the BCAA media content of GW-treated cells, we investigated the effect of GW on BCAA catabolic enzyme expression. To our surprise, GW at 1 *μ*M for either 3, 6, or 24 hours did not alter the mRNA expression of *Bckdha*, *Bcat2*, or the valine-specific catabolic gene 3-hydroxyisobutyrate dehydrogenase (*Hibadh*) (Figure 9(c)). Similarly, no changes were observed in the protein expression of LAT1 (the predominant BCAA transporter), BCKDHa, pBCKDHA, or BCAT2 after 24-hour treatment (Figure 9(d)). Taken together, these findings suggest heightened BCAA uptake (and possibly metabolism) may occur without substantial changes in the protein levels of related cell machinery.

### 3.3. Effect of GW with and without Insulin Resistance on BCAA Catabolic Enzyme Expression and BCAA Media Content

**Figure 10:**
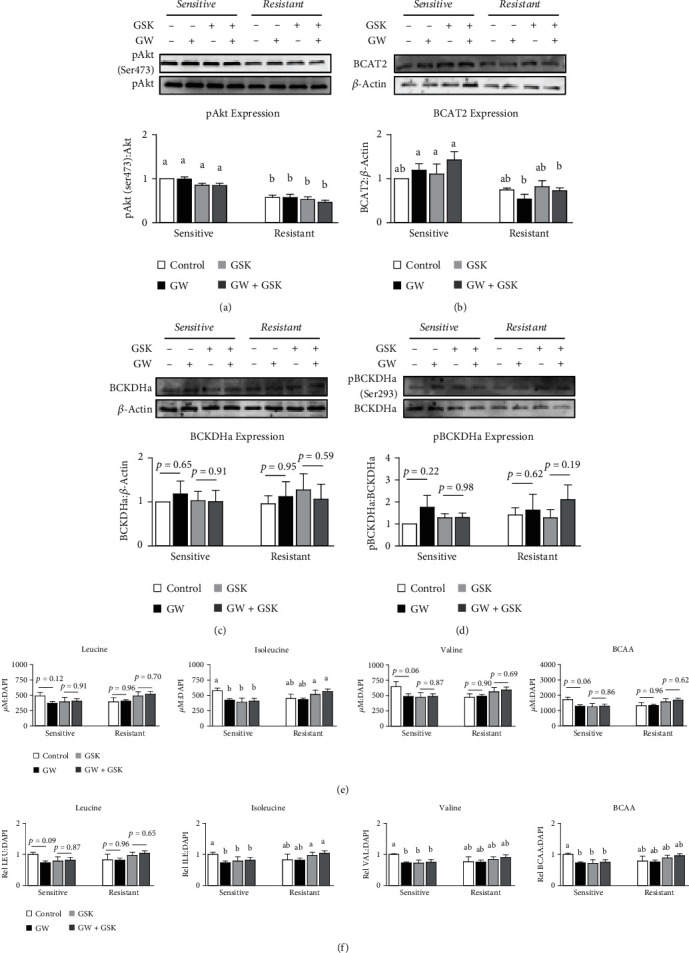
Effect of GW501516 with and without insulin resistance on insulin sensitivity, BCAA catabolism, and extracellular BCAA accumulation. (a, b, c, and d) Effect of GW501516 (GW) at 1 *μ*M for 24 hours both with and without GSK3787 at 10 *μ*M both with and without insulin resistance on (a) insulin sensitivity indicated by pAkt (Ser473):Akt, (b) BCAT2, (c) BCKDHa, and (d) pBCKDHa expression. (e and f) Effect of GW with and without GSK3787 at 10 *μ*M both with and without insulin resistance on (e) individual and cumulative extracellular BCAA normalized to nuclei content (DAPI) or (f) normalized within each independent experiment. Notes: Data were analyzed using factorial ANOVA followed by pair-wise comparisons with Bonferroni's correction for multiple comparisons. Western blots were performed using three replicates per group across two independent experiments with each target quantified in duplicate for each sample, with *n* = 6 for the final analysis. Media analytes were analyzed using three replicates per group across two independent experiments with each metabolite quantified in triplicate for each sample, with *n* = 5–6 for the final analysis. Data with dissimilar letters indicate *p* < 0.05. When significant simple main effects for GW were not observed, exact *p* values are displayed.

Lastly, we reassessed the effect of GW on indicators of BCAA metabolism both with and without insulin resistance, both with and without the PPAR*β*/*δ* inhibitor GSK3787. As expected, insulin resistance was confirmed by reduced pAkt activation following insulin stimulation (Figure 10(a)), though no main effect of GW was observed. Next, we assessed BCAA catabolic enzyme expression and again found no effect of GW on BCAT2, BCKDHa, or pBCKDHa expression (Figures 10(b), 10(c), and 10(d)), though we did observe a main effect of insulin resistance for reduced BCAT2 expression (Figure 10(b)). Lastly, we assessed the effect of each treatment condition on individual and cumulative media BCAA content and found a significant effect of GW for isoleucine when comparing GW to true control, but not leucine, valine, or cumulative media BCAA content (Figure 10(e)). Interestingly, upon further normalization to independent experiments, significant effects of GW were observed for isoleucine, valine, and cumulative BCAA when comparing GW to true control, which was not observed for leucine (Figure 10(f)). Also interesting was the observation that significant simple main effects of GW were only observed when comparing true control and GW-only treated cells (which was also observed in previous experiments, see Figure 9), while GW had no significant effect on BCAA metabolites in any of the other paired comparisons (Figure 10(f)).

## 4. Discussion

PPAR*β*/*δ* regulates several important aspects of muscle energetics and mitochondrial content. Through interactions with PGC-1*α*, PPAR*β*/*δ* activation can enhance mitochondrial content and lipid oxidation. Thus, given the several observations that show PPAR*β*/*δ* can upregulate PGC-1*α*, and the observations that PGC-1*α* (along with PPARs) can regulate BCAA catabolic gene expression, we assessed whether PPAR*β*/*δ* agonism could upregulate BCAA disposal and/or catabolic machinery with GW, a commonly used PPAR*β*/*δ* agonist [1, 25, 27–29, 42, 44, 45, 50]. In line with our original hypothesis, we observed consistently lower BCAA content in the media of GW-treated cells versus control. These findings suggest heightened BCAA uptake (and possibly metabolism) may occur without substantial changes in the protein levels of related catabolic machinery. During follow-up experiments with both insulin sensitive and insulin resistant cells both with and without the presence of a PPAR*β*/*δ* inhibitor, we again found GW lowered BCAA media content in insulin sensitive cells that did not receive the inhibitor (again, independent of change in BCAT2 or BCKDHa), but not in other experimental groups. The implications of these findings are unclear as altered media amino acid content could suggest improved metabolism or simply increased uptake (which were not assessed in this report). Thus, to clarify the true effect of GW on BCAA metabolism, BCAA tracer experiments may be required. That being said, given extracellular BCAA accumulation is the primary correlate of BCAA versus insulin resistance, extracellular BCAA content is an ideal measurement in this context. Additionally, while we did not observe altered BCAA media content during insulin resistance, previous experiments have verified this effect (though in the previous experiments, cells received twice the duration of insulin resistance [41]). Thus, BCAA accumulation in this model of insulin resistance may only occur after more chronic exposure to hyperinsulinemic conditions, which is another variable worthy of further exploration.

Beyond BCAA media content, we also assessed the effect of GW on BCAA catabolic machinery. However, contrary to our hypothesis (and despite lower BCAA media content), PPAR*β*/*δ* agonism with GW did not alter BCAA catabolic enzyme expression at the mRNA or protein level, nor did it alter the activity (indicated by phosphorylation status) of the rate-limiting enzyme complex (BCKDH). We found this surprising because we observed increased PGC-1*α* following GW treatment, and several previous experiments have shown ectopic PGC-1*α* over-expression in C2C12 cells consistently upregulated expression of amino acid metabolism [16, 19], which appears to translate *in vivo* [30]. That being said, the metabolism of BCAA is also dependent on substrate availability (including BCAA, NADH, acyl-CoA, and other) in addition to enzyme abundance and phosphorylation state of the BCKDH complex (details of which are reviewed in detail elsewhere [51]). For example, BT2, an inhibitor of BCKDK, markedly reduced BCKDH complex phosphorylation in various tissues yet only enhanced BCAA metabolism in skeletal muscle [52]. Our findings demonstrate GW-only treated cells exhibited increased BCAA utilization independent of change in BCKDHa phosphorylation, which supports the observation that phosphorylation state of the BCKDH complex is only one of several regulatory mechanisms. Moreover, there are certainly conceivable differences that occur during PGC-1*α* over-expression experiments, which undoubtedly lead to more pronounced activation of several metabolic pathways. And importantly, Kleiner et al. also demonstrated that PPAR*β*/*δ* agonism via GW is dependent on PGC-1*α* abundance [42]. Thus, while we observed significant increases in PGC-1*α* protein content, it could be that the increase was too subtle to elicit some of the anticipated effects of the measured molecular targets. Moreover, several related and alternative targets were not explored by the current report (such as LAT2). Such targets may be relevant as previous experiments have demonstrated a dispensable nature of LAT1, which is compensated for by other transports [53–55]. This example of LAT1 dispensability has been shown in gastrocnemius of LAT1-knockout mice that exhibit increased LAT2 mRNA expression (*Slc7a8*) and do not display altered intra-gastrocnemius leucine content following a fed state (and display elevated leucine content under a fasting state) [55]. Our findings seem to agree with other experiments showing LAT1 abundance is not dependent on cell culture media leucine content in myocytes [56], nor dependent on dietary protein within skeletal muscle [57]. Together, our study provides a first level of evidence suggesting PPAR*β*/*δ* agonism with GW may upregulate BCAA metabolism.

Another important finding worthy of comment was our observation of increased PGC-1*α* protein content that occurred independent of enhanced *Ppargc1a* expression, which is in-line with several other observations [58–61]. Specifically, Luquet et al. showed that despite upregulated indicators of lipid oxidation, muscle-specific PPAR*β*/*δ* overexpression did not alter mRNA expression of *Ppargc1a*, *Tfam*, or *Fat* [61]. Similarly, muscle-specific PPAR*β*/*δ* overexpression performed by Wang et al. demonstrated increased mitochondrial biogenesis independent of changes in *Ppargc1a* expression [60]. Kleiner et al. showed that PPAR*β*/*δ* agonism via GW could enhance primary mouse myotube lipid metabolism, which occurred independent of changes in mRNA expression of regulators of mitochondrial biogenesis and content (*Ppargc1a*, *Ppargc1b*, *Esrra, Cytc*, *Atp5a*, and *Idh3a)* and mitochondrial respiratory protein content [42]. The same report also verified increased indicators of lipid oxidation but not mitochondrial biogenesis in the skeletal muscle of both lean and *ob/ob* mice given GW [42]. Interestingly, the same study also demonstrated the suppression of PGC-1*α* abolished the increased lipid oxidation capacity induced by GW [42]. Other reports using mice with myocardial infarction have also shown GW increases markers of lipid oxidation independent of increased *Ppargc1a* expression [27]. Since these original observations, it has since been established PPAR*β*/*δ* can elevate muscle PGC-1*α* protein content independent of *Ppargc1a* expression [58], which appears to be mediated by PPAR*β*/*δ* suppression of PGC-1*α* protein ubiquitination and degradation [59].

PPAR*β*/*δ* activation via GW has also been shown by some experiments to upregulate PGC-1*α* expression and mitochondrial biogenesis in skeletal muscle both *in vitr*o and *in vivo* [1]. Another report that used a similar model and treatment protocol as the present report showed C2C12 myoblasts treated for 24 hours with 1 *μ*M GW displayed increased PGC-1*α* promoter activity and mRNA expression [25]. Similarly, C2C12 myotubes treated with GW for 24 hours displayed increased PGC-1*α* mRNA [26]. Moreover, treatment with GW and BMS649 (a selective RXR agonist) induced PGC-1*α* expression in C2C12 myocytes [8]. It is unclear why this disparity in *Ppargc1a* expression exists between reports using GW as a PPAR*β*/*δ* agonist; however, it has been shown during experiments in C2C12 cells using GW as a PPAR*β*/*δ* agonist that PPAR response element (PPRE) activation is likely dependent on several factors, including PGC-1*α* abundance and RXR activation [44]. Similar suggestions have been made by other researchers who showed no effect of GW on indicators of mitochondrial biogenesis within a cell culture model of skeletal muscle or in mouse *in vivo* experiments [42].

Other reports have also examined the effect of GW on muscle metabolism. For example, one similar report treated C2C12 cells with 1 *μ*M concentration for 24 hours as performed in the presented experiments. This report showed no effect of GW on basal or peak mitochondrial respiration but showed increased uncoupling with the addition of GW. The report also found elevated levels of *Cpt1* expression, which we did not observe during our experiments at the protein level. Additionally, our report identified a significant increase in basal and peak mitochondrial function as well as mitochondrial content in GW-treated cells. However, an important difference between the two reports was the co-treatment with palmitate performed by Tumova et al., which investigated the involvement of PPAR*β*/*δ* in suppressing the negative effects of palmitate on myocyte survival. Thus, perhaps the effect of GW on expressional profiles is dependent on the availability of other lipids. Moreover, elevated muscle-specific PGC-1*α* overexpression has also been linked with increased lipid content and lipogenic signaling in skeletal muscle of sedentary and exercised mice [62, 63]. This too appears to occur in a way proportional to other signaling molecules (such as SREBP1c and LXR/RXR) [62]. Collectively, induction of PGC-1*α* and activation of PPAR*β*/*δ* may promote increased cellular lipid content (as seen in our report) to support increased oxidative metabolism.

It is also worth highlighting some additional study limitations and considerations. First, although we used a similar treatment protocol to several other experiments [25, 26, 28, 31], we cannot exclude the possibility that our treatment conditions with GW were not optimized to yield comparable results with studies demonstrating increased *Ppargc1a* (optimization that may be required to elicit an observable effect on BCAA catabolic capacity, as well as that of other pathways such as lipid metabolism). However, we did observe increased PGC-1*α* protein content, and thus we suspect that had PPAR*β*/*δ* agonism via GW been sufficient to activate BCAA catabolic machinery, we would have observed some change in the measured indicators. However, it is also conceivable that GW might elicit a different response in the presence of supplemental RXR agonism, various lipids, or in the treatment of diseased cells (versus the otherwise healthy cells used in our experiments). Moreover, while we assessed BCAA concentration in the media (as a surrogate for circulating BCAA blood levels) following treatment and showed differences between groups indicating GW enhances BCAA disposal, we did not vary BCAA levels to determine if GW's effect on BCAA metabolism is dependent on BCAA abundance. Thus, these data should be interpreted with these limitations in mind. Despite these limitations, we feel our report provides an informative overview of the effect of PPAR*β*/*δ* agonism on myotube metabolism and regulation of BCAA metabolism, which collectively suggest PPAR*β*/*δ* agonism via GW increases BCAA disposal in a manner independent of alterations in BCAA catabolic machinery abundance.

## Figures and Tables

**Table 1:** Summary of experimental model, treatment conditions, and related findings to present report.

Model	Dose of GW501516	Duration	Effect	Reference
C2C12 Myotubes	10 nM	16 hours	↑ Glucose uptake	Kramer et al. [29]
	100 nM	24 hours	↑ Cpt1, Pdk4, Ucp3 mRNA	Kleiner et al. [42]
	1 *μ*M	24 hours	↑ Pdk4 mRNA expression	Aoki et al. [43]
	1 *μ*M	24 hours	↑ Cpt1, Pdk4, Ucp3 mRNA and ↔mitochondrial function	Tumova et al. [28]
	1 *μ*M	24 hours	↑ PPAR and PGC-1*α* promoter activity and Ppargc1a and Cpt1 mRNA expression	Hondares et al. [26]
	1 *μ*M	24 hours	↑ PPAR and PGC-1*α* promoter activity and Ppargc1a and Ucp2 mRNA expression	Miura et al. [25]
	1 *μ*M∗	24 hours	↑ PPAR and PGC-1*α* promoter activity and Ucp1, Ucp2, Ucp3 mRNA expression	Dressel et al. [44]
	1 *μ*M + BMS691 at 10 *μ*M	16 hours	↑ Ppargc1a mRNA	Schuler et al. [8]
	1 *μ*M + PAL at 500 *μ*M	16 hours	↑ pAMPK, PDK4, and CPT1 protein expression and rescued palmitate oxidation	Coll et al. [45]
	1 *μ*M	48 hours	↑ PPAR reporter activity	Remels et al. [31]
	5 *μ*M	16 hours	↑ Or rescued Cpt1 mRNA expression/rescued palmitate oxidation (TNF*α* suppressed)	Zizola et al. [27]
	10 *μ*M	24 hours	↑ Cpt-1b, Acadm, and Acox mRNA expression and pAMPK protein expression	Aguilar-Recarte et al. [46]
L6 Myotubes	30, 100, and 300 nM (L6 myotubes)	24 hours	↑ Ppargc1a, Ucp2, Ucp3, and Cpt1 mRNA expression and palmitate oxidation	Tanaka et al. [1]
	100 nM (L6 myotubes)	5 days	↑ UCP3 and ↓GLUT4 protein expression	Higashida et al. [47]
	1 *μ*M (L6 myotubes)	24 hours	↑ PGC-1*α* and CPT1 with ↔ PPAR*δ* protein expression and ↑ palmitate oxidation	Dimopoulos et al. [48]
	1 *μ*M (L6 myotubes)	24 hours	↑ Palmitate oxidation and Cpt1 mRNA expression	Hondares et al. [26]

∗Indicates effects were enhanced by concurrent RXR agonism. Abbreviations: *Cpt1*: carnitine palmitoyl transferase 1; *Glut4*: glucose transporter 4; *Ppargc1a*: peroxisome proliferator-activated receptor gamma coactivator 1-alpha; *Ppar*: peroxisome proliferator-activated receptor; *Ppard*: peroxisome proliferator-activated receptor delta; *Pdk4*: pyruvate dehydrogenase kinase 4; *Ucp*: uncoupling protein.

**Table 2:** Summary of qRT-PCR primers from Integrated DNA Technologies (Coralville, IA).

Gene abbreviation	Forward sequence	Reverse sequence
Atp5b	5′-AGGCCCTTTGCCAAGCTT-3′	5′-TTCTCCTTAGATGCAGCAGAGTACA-3′
Bcat2	5′-CGGACCCTTCATTCGTCAGA-3′	5′-CCATAGTTCCCCCCCAACTT-3′
Bckdha	5′-CCAGGGTTGGTGGGATGAG-3′	5′-GGCTTCCATGACCTTCTTTCG-3′
Cebp	5′-GTGTGCACGTCTATGCTAAACCA-3′	5′-GCCGTTAGTGAAGAGTCTCAGTTTG-3′
Cox5a	5′-GCTGCATCTGTGAAGAGGACAAC-3′	5′-CAGCTTGTAATGGGTTCCACAGT-3′
Cs	5′-TGAGAGGCATGAAGGGACTTGTGT-3′	5′-ATCTGTCCAGTTACCAGCAGCCAA-3′
Fat (CD36)	5′-TAGTAGAACCGGGCCACGTA-3′	5′-CAGTTCCGATCACAGCCCAT-3′
Slc2a4 (Glut4)	5′-GATGAGAAACGGAAGTTGGAGAGA-3′	5′-GCACCACTGCGATGATCAGA-3′
Hibadh	5′-GCAGCGGTGTGTTCTAGGTC-3′	5′-ACACGTCATAGAGGATGAGTGG-3′
Ldha	5′-GGCTTGTGCCATCAGTATCT-3′	5′-CCCGCCTAAGGTTCTTCATTAT-3′
Ldhb	5′-AGTCTCCCGTGCATCCTCAA-3′	5′-AGGGTGTCCGCACTCTTCCT-3′
Nrf1	5′-ACCCTCAGTCTCACGACTAT-3′	5′-GAACACTCCTCAGACCCTTAAC-3′
Pdh	5′-GAAGGCCCTGCATTCAACTTC-3′	5′-ATAGGGACATCAGCACCAGTGA-3′
Ppargc1a	5′-GACAATCCCGAAGACACTACAG-3′	5′-AGAGAGGAGAGAGAGAGAGAGA-3′
Ppara	5′-CTCGCGTGTGATAAAGC-3′	5′-CGATGCTGTCCTCCTTG-3′
Ppard	5′-GCCTCGGGCTTCCACTAC-3′	5′-AGATCCGATCGCACTTCTCA-3′
Pparg	5′-TTCAGCTCTGGGATGACCTT-3′	5′-CGAAGTTGGTGGGCCAGAAT-3′
Scd1	5′-CATCGCCTGCTCTACCCTTT-3′	5′-GAACTGCGCTTGGAAACCTG-3′
Srebp1	5′-ATCGCAAACAAGCTGACCTG-3′	5′-AGATCCAGGTTTGAGGTGGG-3′
Tbp	5′-GGGATTCAGGAAGACCACATA-3′	5′-CCTCACCAACTGTACCATCAG-3′
Tfam	5′-GAAGGGAATGGGAAAGGTAGAG-3′	5′-ACAGGACATGGAAAGCAGATTA-3′

Abbreviations: *Atp5b*: ATP synthase F1; *Bcat2*: branched-chain aminotransferase 2, *Bckdha*: branched-chain alpha-keto acid dehydrogenase; *Cebpa*: CCAAT/enhancer-binding protein alpha; *Cox5a*: cytochrome C oxidase Subunit 5A; *Cs*: citrate synthase; *Fat* or *CD36*: fatty acid translocase; *Slc2a4* or *Glut4*: glucose transporter 4; *Hibadh*: 3-hydroxyisobutyrate dehydrogenase; *Ldha*: lactate dehydrogenase a; *Ldhb*: lactate dehydrogenase b; *Nrf1*: nuclear respiratory factor 1; *Pdh*: pyruvate dehydrogenase; *Ppargc1a*: peroxisome proliferator-activated receptor gamma coactivator 1-alpha; *Pk1*: pyruvate kinase 1; *Ppara*: peroxisome proliferator-activated receptor alpha; *Ppard*: peroxisome proliferator-activated receptor delta; *Pparg*: peroxisome proliferator-activated receptor gamma; *Scd1*: stearoyl-CoA desaturase; *Srebp1*: sterol regulatory element-binding protein; *Tbp*: TATA box binding protein; and *Tfam*: mitochondrial transcription factor A.

**Table 3:** Summary of primary antibodies used for western blot experiments.

Protein target	Type	Dilution	Company	Item	Approx. Mol Wt.	Product link
ACAD9	RP	1 : 1000	SC Biotechnology	sc-135148	68kd	Datasheet
pAkt (Ser 473)	RP	1 : 1000	SC Biotechnology	sc-7985-R	62kd	p-Akt1/2/3 (Ser 473)
Akt	RP	1 : 1000	Cell Signaling	9272	62kd	Akt Antibody#9272
*β*-Actin	RP	1 : 1000	SC Biotechnology	sc-130656	43kd	Datasheet
BCAT2	RP	1 : 1000	Bioss	BS-6589R	44kd	Datasheet
BCKDHa	RP	1 : 1000	ProSci	31-325	50kd	BCKDHA Antibody
pBCKDHa (Ser 293)	RP	1 : 1000	AbCam	ab200577	50kd	Phospho BCKDHA (S293)
CPT1b	RP	1 : 1000	SC Biotechnology	sc-20670	75kd	Datasheet
CS	MM	1 : 1000	SC Biotechnology	sc-390693	52kd	sc-390693
ERR*α*	RP	1 : 1000	SC Biotechnology	sc-66882	53kd	Datasheet
LAT1	RP	1 : 500	NOVUS Biological	NBP3-09988	47kd	Datasheet
NRF1	RP	1 : 1000	SC Biotechnology	sc-33771	68kd	Datasheet
PGC-1*α*	RP	1 : 1000	SC Biotechnology	sc-13067	90kd	PGC-1α (H-300)
PPAR*β*/*δ*	RP	1 : 1000	SC Biotechnology	sc-7197	52kd	Datasheet
PPAR*γ*	RP	1 : 1000	SC Biotechnology	sc-7196	52kd	Datasheet
SREBP1	RP	1 : 1000	SC Biotechnology	sc-366	68kd	Datasheet
TFAM	RP	1 : 1000	SC Biotechnology	sc-28200	25kd	Datasheet
UCP1/2/3	RP	1 : 1000	SC Biotechnology	sc-28766	33kd	Datasheet

Abbreviations: ACAD9: Acyl-coA dehydrogenase 9; BCAT2: branched-chain aminotransferase 2; BCKDHE1*α*: branched-chain alpha-keto acid dehydrogenase E1*α*; CPT1b: carnitine palmitoyl transferase; CS: citrate synthase; ERR*α*: estrogen-related receptor alpha; LAT1: large amino acid transporter 1; MM: mouse monoclonal; NRF1: nuclear respiratory factor 1; PGC-1*α*: peroxisome proliferator-activated receptor gamma coactivator 1-alpha; PPAR*β*/*δ*: peroxisome proliferator-activated receptor beta/delta; PPAR*γ*: peroxisome proliferator-activated receptor gamma; SREBP1: sterol regulatory element-binding proteins; RP: rabbit polyclonal; TFAM: mitochondrial transcription factor A; and UCP: uncoupling protein 1/2/3. Notes: Target molecular weight was based on product datasheet. Molecular weights for all targets were verified against sizes suggested by product brochures.

## Data Availability

The data that support the findings of this study are available from the corresponding author upon reasonable request.

## Abbreviations

AMPK- 5′: AMP-activated protein kinase
*Atp5b*: ATP synthase (mRNA transcripts from *Atp5b* gene)
BCAA: Branched-chain amino acid
*Bcat2*: Branched-chain amino acid transaminase 2 (mRNA)
BCAT2: Branched-chain amino acid transaminase 2 (protein)
*Bckdh*: Branched-chain alpha-keto acid dehydrogenase (mRNA)
BCKDH: Branched-chain alpha-keto acid dehydrogenase (protein)
BCKDK: Branched-chain alpha-keto acid dehydrogenase kinase (protein)
*Cox5a*: Cytochrome c oxidase (mRNA transcripts from *Cox5a*)
*Cpt1*: Carnitine palmitoyl transferase 1 (mRNA)
CPT1b: Carnitine palmitoyl transferase 1 b (protein)
*Cs*: Citrate synthase (mRNA)
CS: Citrate synthase (protein)
ECAR: Extracellular acidification rate
ERR*α*: Estrogen-related receptor alpha (protein)
*Esrra*: Estrogen-related receptor alpha (mRNA)
FCCP: Carbonyl cyanide p-[trifluoromethoxy]-phenyl-hydrazone
GLUT4: Glucose transporter 4 (protein)
GW: GW501516
*Hibadh*: 3-hydroxyisobutyrate dehydrogenase (mRNA)
*Slc7a5*: Large amino acid transporter 1 (mRNA)
LAT1: Large amino acid transporter 1 (protein)
*Slc7a8*: Large amino acid transporter 2 (mRNA)
LAT2: Large amino acid transporter 2 (protein)
*Ldha*: Lactate dehydrogenase A (mRNA)
*Ldhb*: Lactate dehydrogenase B (mRNA)
mtDNA: Mitochondrial DNA
*Nrf1*: Nuclear respiratory factor 1 (mRNA)
NRF1: Nuclear respiratory factor 1 (protein)
OCR: Oxygen consumption rate
PGC-1*α*: Peroxisome proliferator-activated receptor gamma coactivator 1-alpha (protein)
*Ppargc1a*: Peroxisome proliferator-activated receptor gamma coactivator 1-alpha (mRNA)
*Ppara*: Peroxisome proliferator-activated receptor alpha (mRNA)
PPAR*α*: Peroxisome proliferator-activated receptor alpha (protein)
*Ppard*: Peroxisome proliferator-activated receptor beta (mRNA)
PPAR*β*: Peroxisome proliferator-activated receptor beta (protein)
*Pk1*: Pyruvate kinase 1 (mRNA)
*Pdh*: Pyruvate dehydrogenase (mRNA)
*Sirt1*: Sirtuin 1 (mRNA)
SIRT1: Sirtuin 1 (protein)
*Sirt3*: Sirtuin 3 (mRNA)
SIRT3: Sirtuin 3 (protein)
*Tbp*: TATA binding protein (mRNA)
*Tfam*: Mitochondrial transcription factor A (mRNA)
TFAM: Mitochondrial transcription factor A (protein).
